# Supplementary material for: The active layer soils of Greenlandic permafrost areas can function as important sinks for volatile organic compounds
Source: Commun Earth Environ. 2025 Jan 17;6(1):32. doi: 10.1038/s43247-025-02007-8 (PMC11748482; doi:10.1038/s43247-025-02007-8)
Supplement: Supplementary file 1 — Supplementary Information [file 43247_2025_2007_MOESM1_ESM.pdf]

Supplementary Information for

The active layer soils of Greenlandic permafrost areas can function as important sinks for volatile organic compounds

Yi Jiao[1,2,*], Magnus Kramshøj[2], Cleo L. Davie-Martin[2,3], Bo Elberling[4], Riikka Rinnan[1,2,*]

1. Center for Volatile Interactions (VOLT), Department of Biology, University of Copenhagen, Universitetsparken 15, Copenhagen Ø, 2100, Denmark

2. Terrestrial Ecology Section, Department of Biology, University of Copenhagen, Universitetsparken 13A, Copenhagen Ø, 2100, Denmark

3. The Climate and Environmental Research Institute (NILU), The Fram Centre, Tromsø, 9296, Norway

4. Department of Geosciences and Natural Resource Management, University of Copenhagen, Øster Voldgade 10, Copenhagen K, 1350, Denmark

* Correspondence should be addressed to Yi Jiao (Email: jiaoyi.joey@gmail.com; yi.jiao@bio.ku.dk) or Riikka Rinnan (E-mail: riikkar@bio.ku.dk)

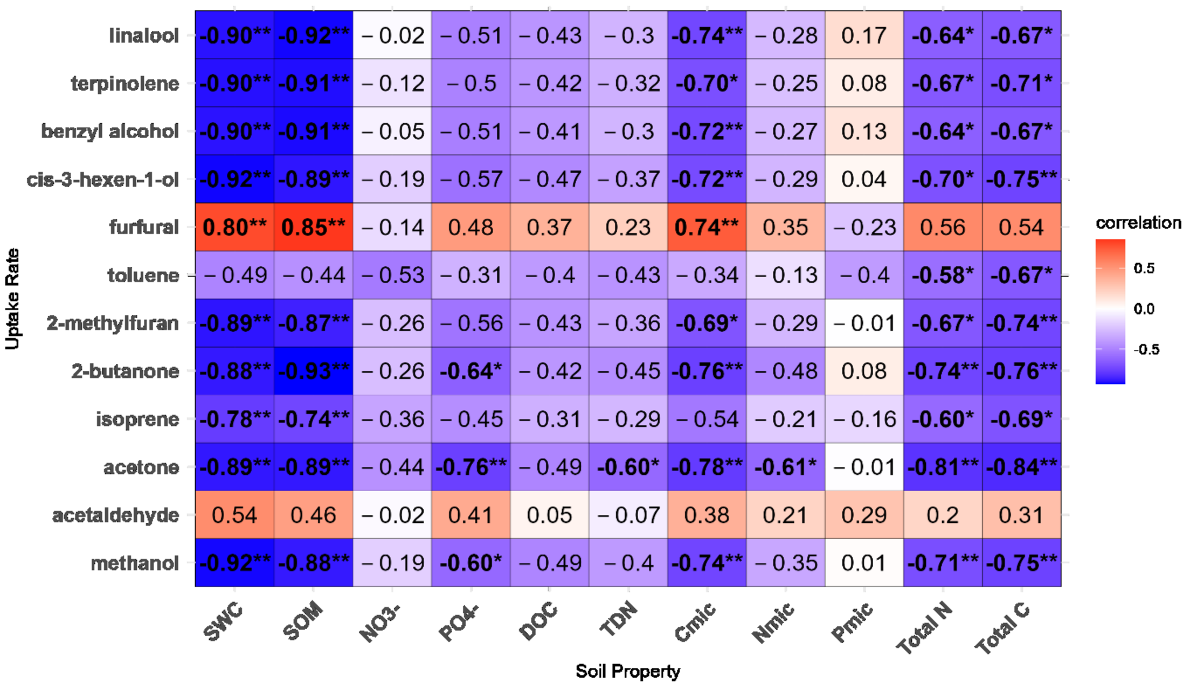

Supplementary Fig. 1 Heatmap showing correlation coefficients between the uptake rates of the VOCs and the soil properties. Heatmap was colored to indicate the direction of the correlation, with red representing positive correlations and blue representing negative correlations; number in each grid cell shows the Pearson's correlation coefficient, labeled with * for significance at $P < 0.05$ and ** for significance at $P < 0.01$; SWC, gravimetric soil water content; SOM, soil organic matter; DOC, dissolved organic carbon; TDN, total dissolved nitrogen; Cmic, microbial biomass carbon; Nmic, microbial biomass nitrogen; Pmic, microbial biomass phosphorus.

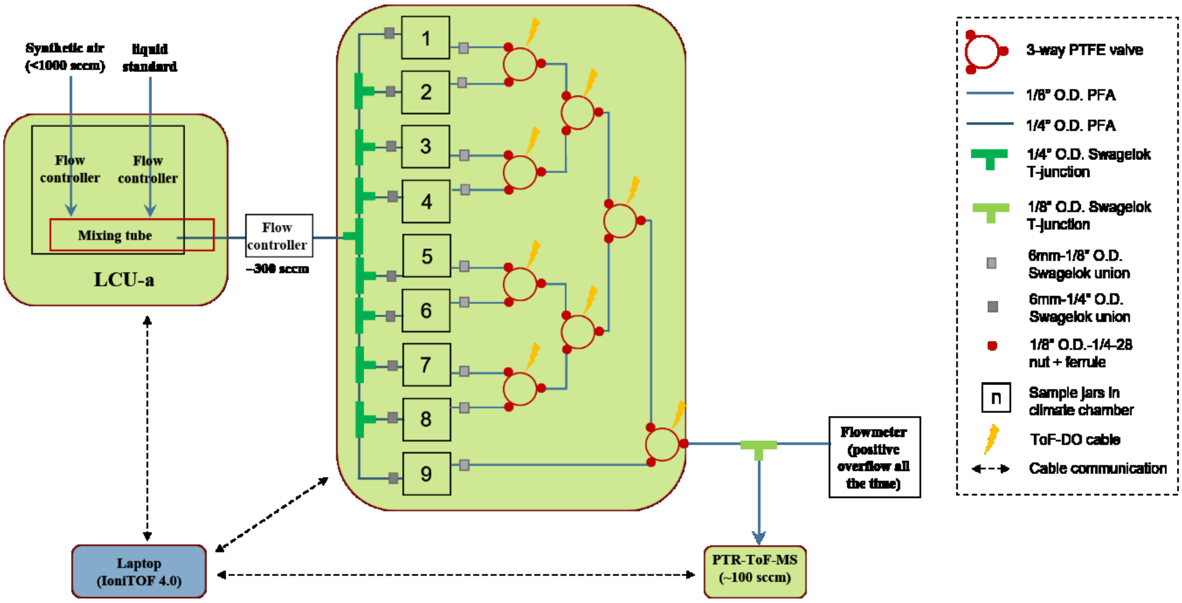

Supplementary Fig. 2 Schematic drawing of the experimental setup. LCU-a – liquid control unit, sccm – standard cubic centimeter per minute, O.D. – outer diameter, PTFE – polytetrafluoroethylene, PFA – perfluoroalkoxy alkane.

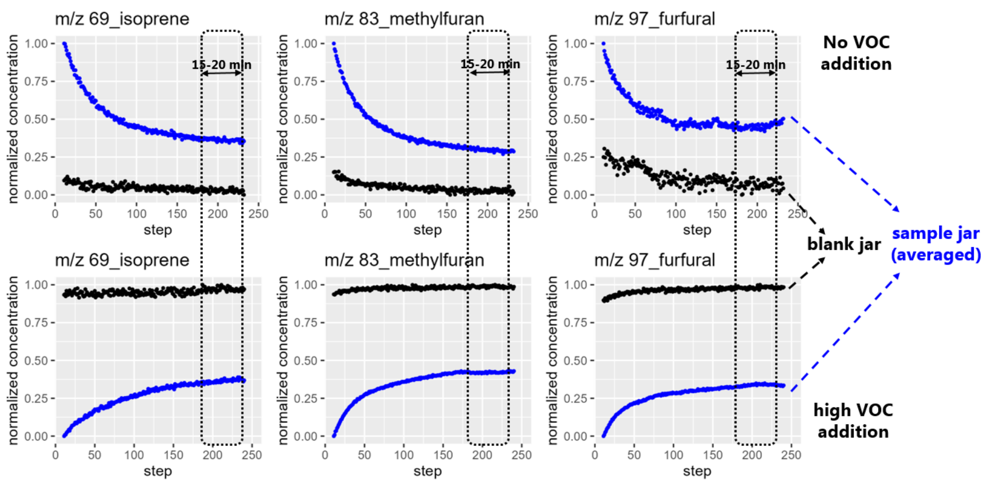

Supplementary Fig. 3: Selected VOC concentration in the outlet gas over time during the experiment. The plots show normalized comparisons between randomly selected sample jars (with soils) and blank jars, under conditions with VOCs amended into the inlet gas (top panel) and without VOC amendments (bottom panel). A step in the X axis represents 5 seconds.

Supplementary Table S1 Emission rates (mean ± S.E., unit: nmol g dw^{-1} hr^{-1}) of the top 10 masses emitted by the soils collected from the active layer at Disko Island (n = 6) and Kangerlussuaq (n = 3) under the VOC-free headspace. A full mass list of the tentative compounds emitted by these soils, along with their emission rates, is available with DOI: 10.5281/zenodo.14185189.

Top 10 protonated mass	Disko Island soil (depth)		
	10 cm	20cm	40cm
**79.056**	6.24 ± 0.81	6.12 ± 0.76	6.16 ± 0.76
**92.061**	3.98 ± 0.50	3.91 ± 0.48	3.93 ± 0.47
**79.077**	3.18 ± 0.37	3.12 ± 0.36	3.15 ± 0.33
**107.053**	3.07 ± 0.38	2.97 ± 0.35	2.99 ± 0.33
**108.057**	2.01 ± 0.27	1.98 ± 0.26	1.97 ± 0.27
**81.071**	2.38 ± 0.48	2.46 ± 0.46	2.38 ± 0.53
**61.034**	1.25 ± 0.30	0.79 ± 0.34	1.12 ± 0.30
**34.025**	1.27 ± 0.27	1.34 ± 0.24	1.36 ± 0.40
**137.133**	1.02 ± 0.14	1.11 ± 0.14	1.04 ± 0.19
**83.080**	0.78 ± 0.05	0.90 ± 0.06	0.75 ± 0.11

Top 10 protonated mass	Kangerlussuaq soils (depth and site)								
	10 cm			20cm			40cm		
	coast	intermediate	glacier	coast	intermediate	glacier	coast	intermediate	glacier
**73.938**	5.41 ± 0.18	5.14 ± 0.79	2.08 ± 1.98	5.41 ± 0.27	5.80 ± 0.38	0.27 ± 0.10	5.90 ± 0.49	9.79 ± 2.55	1.74 ± 1.30
**81.071**	3.31 ± 0.00	3.31 ± 0.00	2.21 ± 1.10	3.31 ± 0.00	3.31 ± 0.00	3.32 ± 0.01	3.31 ± 0.00	3.30 ± 0.02	3.31 ± 0.01
**51.014**	2.05 ± 0.19	3.13 ± 0.16	3.73 ± 0.62	2.30 ± 0.42	3.38 ± 0.73	4.27 ± 0.07	2.25 ± 0.17	3.10 ± 0.60	3.81 ± 0.29
**92.061**	3.81 ± 0.15	2.99 ± 0.12	4.29 ± 0.25	3.74 ± 0.15	3.81 ± 0.58	4.10 ± 0.12	3.86 ± 0.22	3.88 ± 0.50	3.85 ± 0.36
**79.056**	1.85 ± 0.00	1.83 ± 0.00	1.23 ± 0.62	1.84 ± 0.00	1.84 ± 0.00	1.85 ± 0.00	1.84 ± 0.01	1.84 ± 0.00	1.85 ± 0.00
**49.943**	1.60 ± 0.13	1.67 ± 0.10	1.86 ± 0.10	1.66 ± 0.19	1.92 ± 0.26	1.86 ± 0.02	1.41 ± 0.17	1.94 ± 0.19	1.90 ± 0.20
**165.046**	0.77 ± 0.43	0.59 ± 0.08	1.47 ± 0.55	0.65 ± 0.51	0.48±0.12	1.09 ± 0.06	0.76 ± 0.40	0.46 ± 0.06	1.05 ± 0.16
**61.034**	0.65 ± 0.09	2.05 ± 1.52	1.93 ± 0.50	0.57 ± 0.15	1.56 ± 0.82	3.74 ± 1.78	1.49 ± 0.89	5.02 ± 1.00	1.68 ± 0.80
**137.133**	1.57 ± 0.00	1.57 ± 0.00	1.04 ± 0.52	1.57 ± 0.00	1.56 ± 0.00	1.57 ± 0.00	1.57 ± 0.01	1.57 ± 0.00	1.57 ±0.00
**107.986**	2.20 ± 0.02	2.04 ± 0.14	2.63 ± 0.42	2.23 ± 0.08	2.33 ± 0.30	2.33 ± 0.05	2.37 ± 0.21	2.35 ± 0.35	2.21 ± 0.15
