# Supplementary material for: The active layer soils of Greenlandic permafrost areas can function as important sinks for volatile organic compounds
Source: Commun Earth Environ. 2025 Jan 17;6(1):32. doi: 10.1038/s43247-025-02007-8 (PMC11748482; doi:10.1038/s43247-025-02007-8)
Supplement: Supplementary file 2 — Reporting Summary [file 43247_2025_2007_MOESM2_ESM.pdf]

nature portfolio

Corresponding author(s): Riikka Rinnan

Last updated by author(s): Dec 20, 2024

Reporting Summary

Nature Portfolio wishes to improve the reproducibility of the work that we publish. This form provides structure for consistency and transparency in reporting. For further information on Nature Portfolio policies, see our Editorial Policies and the Editorial Policy Checklist.

Statistics

For all statistical analyses, confirm that the following items are present in the figure legend, table legend, main text, or Methods section.

n/a	Confirmed	
☐	☒	The exact sample size (*n*) for each experimental group/condition, given as a discrete number and unit of measurement
☐	☒	A statement on whether measurements were taken from distinct samples or whether the same sample was measured repeatedly
☐	☒	The statistical test(s) used AND whether they are one- or two-sided<br>*Only common tests should be described solely by name; describe more complex techniques in the Methods section.*
☐	☒	A description of all covariates tested
☐	☒	A description of any assumptions or corrections, such as tests of normality and adjustment for multiple comparisons
☐	☒	A full description of the statistical parameters including central tendency (e.g. means) or other basic estimates (e.g. regression coefficient) AND variation (e.g. standard deviation) or associated estimates of uncertainty (e.g. confidence intervals)
☒	☐	For null hypothesis testing, the test statistic (e.g. *F*, *t*, *r*) with confidence intervals, effect sizes, degrees of freedom and *P* value noted<br>*Give P values as exact values whenever suitable.*
☒	☐	For Bayesian analysis, information on the choice of priors and Markov chain Monte Carlo settings
☒	☐	For hierarchical and complex designs, identification of the appropriate level for tests and full reporting of outcomes
☒	☐	Estimates of effect sizes (e.g. Cohen's *d*, Pearson's *r*), indicating how they were calculated

Our web collection on statistics for biologists contains articles on many of the points above.

Software and code

Policy information about availability of computer code

Data collection	The VOC measurements were conducted by a PTR-ToF-MS (TOF-1000 ultra, Ionicon Analytik, Innsbruck, Austria) coupled with a liquid calibration unit (LCU-a, Ionicon Analytik, Innsbruck, Austria) controlled by the software (IoniTOF 4.0, Ionicon Analytik, Innsbruck, Austria). Soil properties were measured using a TOC-L total organic carbon analyzer (Shimadzu, Kyoto, Japan), and an FIA STAR 5000 flow injection analyzer (FOSS Tecator, Höganäs, Sweden).
Data analysis	Raw VOC data (.h5 file) was first processed by the software PTRwid. Flux calculation and statistical analysis were conducted with IBM SPSS Statistics (version 29.0.2.0), SIMCA (version 17.0, Umetrics, Umeå, Sweden), and microsoft Excel (2016).

For manuscripts utilizing custom algorithms or software that are central to the research but not yet described in published literature, software must be made available to editors and reviewers. We strongly encourage code deposition in a community repository (e.g. GitHub). See the Nature Portfolio guidelines for submitting code & software for further information.

Data

Policy information about availability of data

All manuscripts must include a data availability statement. This statement should provide the following information, where applicable:

- Accession codes, unique identifiers, or web links for publicly available datasets
- A description of any restrictions on data availability
- For clinical datasets or third party data, please ensure that the statement adheres to our policy

All data that support the findings of this study is presented in the manuscript, the supplemental material and dataset (https://doi.org/10.5281/zenodo.14185189).

Human research participants

Policy information about studies involving human research participants and Sex and Gender in Research.

Reporting on sex and gender	Not applicable
Population characteristics	Not applicable
Recruitment	Not applicable
Ethics oversight	Not applicable

Note that full information on the approval of the study protocol must also be provided in the manuscript.

Field-specific reporting

Please select the one below that is the best fit for your research. If you are not sure, read the appropriate sections before making your selection.

☐ Life sciences ☐ Behavioural & social sciences ☒ Ecological, evolutionary & environmental sciences

For a reference copy of the document with all sections, see nature.com/documents/nr-reporting-summary-flat.pdf

Ecological, evolutionary & environmental sciences study design

All studies must disclose on these points even when the disclosure is negative.

Study description	To assess the role of the active layer soil in biogeochemical cycling of VOCs, we collected soil samples from contrasting locations in Greenland at different depths. These samples were subjected to controlled laboratory incubations to measure net VOC exchange rates under various environmental conditions. By exposing the soil samples to predetermined VOC mixtures at parts per billion levels, we assessed the potential consumption rates of these compounds in the active layer soils. Particularly, our study was designed to test three hypotheses. First, we expected that the active layer soils possess the capacity for VOC uptake, especially in the upper horizon with the highest microbial biomass and activity. Second, we hypothesized that the older, coastal soils, which likely host more developed, diverse microbial communities than soils close to the ice sheet, would demonstrate a higher and more consistent consumption rate of different VOCs. Third, we expected that the uptake of VOCs is influenced by soil water content, which will change drastically under the future climate. By testing these hypotheses, this study aims to enhance our understanding of the role of the active layer in the biogeochemistry of VOCs in permafrost regions under climate change.
Research sample	The active layer soil samples were collected from two locations in western Greenland: Disko Island and Kangerlussuaq.
Sampling strategy	At Disko Island (Blæsedalen, 69.28°N, 53.48°W), soil was sampled from three depths (10 cm, 20 cm, and 40 cm) at six independent soil pits within an area of 15 x 15 meters, in connection with the establishment of a winter warming experiment. At Kangerlussuaq, soil samples were collected from three sites according to their relative proximity to the continental glacier: glacier site (67.12°N, 50.16°W, closest to the continental glacier), intermediate site (67.06°N, 50.46°W, intermediate), and coast site (67.04°N, 50.55°W, furthest away from the glacier, near coast of Kangerlussuaq Fjord). Within each location, triplicate soil samples (n = 3) were collected at three different depths (10, 20 and 40 cm) from soil pits using stainless steel density ring of approximately 100cm.
Data collection	The VOC measurements were conducted by a PTR-ToF-MS (TOF-1000 ultra, Ionicon Analytik, Innsbruck, Austria) coupled with a liquid calibration unit (LCU-a, Ionicon Analytik, Innsbruck, Austria) controlled by the software (IoniTOF 4.0, Ionicon Analytik, Innsbruck, Austria). Soil properties were measured using a TOC-L total organic carbon analyzer (Shimadzu, Kyoto, Japan), and an FIA STAR 5000 flow injection analyzer (FOSS Tecator, Höganäs, Sweden).
Timing and spatial scale	The samples were analyzed within three month after collection. The spatial scale can be found from their coordinates in the sampling strategy section.
Data exclusions	No data excluded.

Reproducibility	The instruments used in the experiments are all commercially available. We have provided detailed descriptions of the sample collection, experimental setup, and instrument analysis in the accompanying publication. These measures aim to ensure maximum reproducibility of the study.
Randomization	Randomization was applied to ensure unbiased data collection and analysis. Samples were randomized during the experimental setup to avoid systematic errors and ensure representative results. For analyses, the order of measurements was randomized to minimize potential biases.
Blinding	Blinding was not applied in this study, as the experimental setup relied on automated, objective measurements that minimized bias. However, efforts were made to standardize procedures to ensure consistency across all samples.

Did the study involve field work? ☒ Yes ☐ No

Field work, collection and transport

Field conditions	At Disko Island (Blæsedalen, 69.28°N, 53.48°W), the region experiences an Arctic maritime climate, with an annual average air temperature of -3.0 ± 1.8 °C and precipitation of 418 ± 131 mm. The primary ecosystem within this valley is characterized by mesic tundra heath, predominantly featuring both evergreen and deciduous dwarf shrubs, including Betula nana L., Empetrum nigrum ssp. hermaphroditum Hagerup, Cassiope tetragona (L.) D. Don, Salix glauca L., and Vaccinium uliginosum L. These are interspersed with mosses such as Tomentypnum nitens (Hedw.) Loeske, Racomitrium lanuginosum (Hedw.) Brid., Sphagnum spp., and various lichens28. Soil was sampled from three depths (10 cm, 20 cm, and 40 cm) at six independent soil pits within an area of 15 x 15 meters, in connection with the establishment of a winter warming experiment. At Kangerlussuaq, soil samples were collected from three sites according to their relative proximity to the continental glacier: glacier site (67.12°N, 50.16°W, closest to the continental glacier), intermediate site (67.06°N, 50.46°W, intermediate), and coast site (67.04°N, 50.55°W, furthest away from the glacier, near coast of Kangerlussuaq Fjord).
Location	The active layer soil samples were collected from two locations in western Greenland: Disko Island and Kangerlussuaq.
Access & import/export	Samples were collected, transported, and analyzed within the Kingdom of Denmark.
Disturbance	Soil samples were transported back to Copenhagen in plastic bags at temperatures around 5-7 °C and were subsequently stored at -20 °C until the start of the experiments (within three months). In the laboratory, stones, roots, or visible litter were removed from the soils, which were then homogenized and sieved through a 5 mm mesh. By this, we aimed to exclude the potential interferences originating from the heterogeneity of soils, which allowed us to investigate the processes of VOC production and degradation under a controlled experimental setup. In the laboratory, stones, roots, or visible litter were removed from the soils, which were then homogenized and sieved through a 5 mm mesh. By this, we aimed to exclude the potential interferences originating from the heterogeneity of soils, which allowed us to investigate the processes of VOC production and degradation under a controlled experimental setup.

Reporting for specific materials, systems and methods

We require information from authors about some types of materials, experimental systems and methods used in many studies. Here, indicate whether each material, system or method listed is relevant to your study. If you are not sure if a list item applies to your research, read the appropriate section before selecting a response.

Materials & experimental systems

n/a	Involved in the study	
☒	☐	Antibodies
☒	☐	Eukaryotic cell lines
☒	☐	Palaeontology and archaeology
☒	☐	Animals and other organisms
☒	☐	Clinical data
☒	☐	Dual use research of concern

Methods

n/a	Involved in the study	
☒	☐	ChIP-seq
☒	☐	Flow cytometry
☒	☐	MRI-based neuroimaging
